# Prostate Cancer in the Caribbean

**DOI:** 10.7759/cureus.50150

**Published:** 2023-12-08

**Authors:** Nuneaton S Ramesar, Amalia Hosein, Kristy Samaroo, Jameel Ali

**Affiliations:** 1 General Practice/Public Health/Occupational Health, The University of Trinidad and Tobago, Port of Spain, TTO; 2 Biomedical Engineering, The University of Trinidad and Tobago, Port of Spain, TTO; 3 Surgery, University of Toronto, Toronto, CAN

**Keywords:** prostate cancer risks, prostate disease, cancer epidemiology, genetic epidemology, main male cancers, non-genetic risks, epidemiology, oncology, genetics, prostate cancer research

## Abstract

Prostate cancer (PC) is one of the principal causes of cancer death worldwide. The mortality rate for PC in the Caribbean is higher than in many developed countries, and there is a difference in the incidence among the various Caribbean nations. Besides surveillance and screening, these factors increase concerns about genetic and other risk factors causing PC incidence. PC research is limited in scope and regularity in the Caribbean, creating a literature gap. This literature review aims to examine the PC situation in the Caribbean to highlight where further studies are needed. This review includes all available studies on PC in the specified Caribbean population from 1958 to 2023 utilising the keywords “Prostate Cancer and Caribbean” on PubMed, Scopus, and ScienceDirect databases. The information is then structured by Caribbean countries and by seven themes. These themes are PC incidence and mortality, demographics, clinicopathology, genetics, non-genetic risks, diagnosis and treatment, and PC control. The findings demonstrated that countries with low resources are burdened by more severe illnesses with worse PC outcomes. Furthermore, territories with national cancer registries seemed to have enhanced methods for PC management. In conclusion, this review is significant because it provides initial support for researchers, administrators, and planners for PC healthcare. Additionally, it gives an opportunity for further epidemiological analyses that can supply more significant insights into the PC situation in the Caribbean. Further research should focus on prevention strategies and the standardisation of treatment procedures to enhance surveillance and improve patient outcomes.

## Introduction and background

Prostate cancer (PC) is the fifth highest cause of cancer death affecting men worldwide in 2020 [1]. One of the highest mortality rates for PC is in the Caribbean, surpassing various developed countries, 75.8 per 100,000 people [2]. Furthermore, there is a disparity in incidence among the different Caribbean countries [3]. This situation raises concerns about genetic and other risk factors causing PC incidence, apart from surveillance and screening.

African descendants from Central and West Africa constitute more than 90% of the Caribbean population. From this population, Afro-Caribbean men have an extremely higher risk of PC than any other men worldwide [3]. Comprehending fully the genetic and other risk factors for PC in the Caribbean is critical for understanding and planning approaches to PC management.

Occasionally, specific risk factors interact with genetic variants to affect PC overall risk and prognosis [4]. Knowledge of these factors could lead to modification of screening and therapy for high-risk Caribbean men. Consequently, public health policies could be altered, health outcomes of men enhanced, and the burden of PC decreased.

This literature review assessed the PC situation in the Caribbean to emphasise where further studies may be required. All accessible publications were reviewed within the last 65 years (1958 to 2023) using the search words “Prostate Cancer and Caribbean” on PubMed, Scopus, and ScienceDirect databases. The data were organized regionally by Caribbean country and thematically utilising seven themes. These themes are PC incidence and mortality, demographics, clinicopathology, genetics, risk factors, diagnosis and treatment, and PC control.

## Review

Methodology

**Figure 1:**
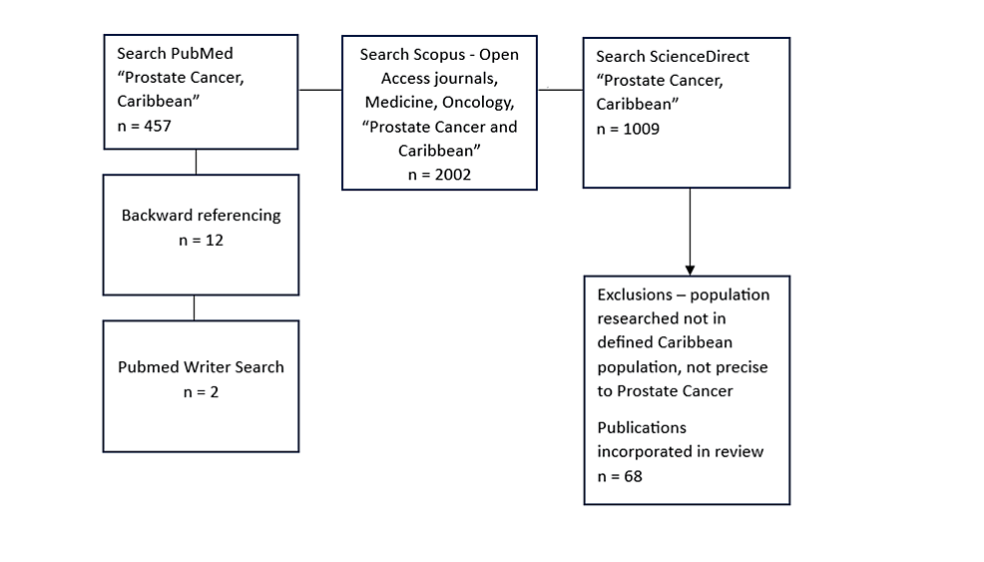
Flow chart for methodology

This review incorporated all articles from 1958 to 2023 after searching databases PubMed, Scopus, and ScienceDirect utilising essential search words “Prostate Cancer and Caribbean”. Additionally, the references from many publications were examined to identify further articles that could not be accessible on PubMed. Significant writers discovered were explored on PubMed to recognise missed articles. Furthermore, Scopus included filters that comprised Display only Open Access Journals, and Subject areas with Medicine and Oncology. Full texts with abstracts were examined for inclusion eligibility. All research concentrated on PC in the specified Caribbean population (Appendix) and studies with comparisons with people from other parts of the world and immigrant Caribbean people. Lastly, 68 articles that met the inclusion criteria were included as depicted in Figure 1.

The information was structured and displayed under the following headlines and stated by country as the accessible literature allowed: PC incidence and mortality, demographics, clinicopathology, genetics, risk factors, diagnosis and treatment, and PC control. Furthermore, the number of available publications is presented in Table 1.

**Table 1:** Published articles on prostate cancer (PC) for incidence, mortality, demographics, clinicopathology, genetics, risk factors, diagnosis and treatment, and control for each Caribbean territory

Caribbean territory	No. of articles	Incidence	Mortality	Patient demographics	Clinicopathology	Genetics	Risk factors	Diagnosis and Treatment	PC Control
Antigua and Barbuda	3	X	X	X	X			X	X
The Bahamas	5	X	X	X	X			X	X
Barbados	5	X	X	X	X		X		
Cuba	6	X	X	X			X	X	X
Dominica	2	X	X	X					
French West Indies (French Guiana, Guadeloupe, and Martinique	8	X	X	X	X	X	X	X	X
Grenada	2	X	X	X					
Guyana	3	X	X	X					
Haiti	2	X	X	X					
Jamaica	14	X	X	X	X	X	X	X	X
The Netherlands Antilles	2	X	X						
St. Kitts and Nevis	1	X	X						
St. Vincent and Grenadines	1	X	X						
Suriname	1	X	X	X					
Trinidad and Tobago	13	X	X	X	X	X	X	X	X

Prostate cancer incidence and mortality

Twelve Caribbean countries have cancer registries with four supplying incidence data of high quality covering 14.4% of the population in the Caribbean region [5,6]. Considering the small number of countries with high-quality incidence data, evaluating PC incidence and mortality in this region is difficult [5].

Antigua and Barbuda

From January 1, 2001, to December 31, 2005, 492 cancer cases were reported after histological verification with 108 PC having an age standardised rate (ASR) of 69.4 incidences per 100,000 [7] period noted 51.9% of male cancer deaths at a mean age of 79, with a mortality of 57.2 deaths per 100,000 [8]. Comparing the data from the 1984 to 1989 research by Simon et al. [7] demonstrated a significant increase in crude mortality rate due to PC (ASR 57 vs 13) [8].

The Bahamas

In 15 years from 1988 to 2002, the Grand Bahama island had an incidence cancer rate per year ranging from 57.4 to 108.6 per 100,000 approximately with male 59.2 versus female 109.8 per 100,000. The most common male cancer was prostate 41 per 100,000. For this period, the ASR per 100,000 for annual cancer mortality was 114.8 (140.2 for males and 103.3 for females). Furthermore, PC accounted for 14.5% (52) of total cancer deaths (359) and 28.7% of male cancer deaths (181) [9].

Barbados

Standardised to the United States (US) population, 1,101 new PC cases were found in Barbados for an incidence rate of 160.4 per 100,000 from July 2002 to December 2008. All these cases were confirmed histologically. Subsequently, deaths registered from PC were investigated over 14 years beginning in January 1995. PC mortality rates fluctuated from 63.2 to 101.6 per 100,000, which seemed higher than 51.1 to 78.8 per 100,000 among African Americans [10].

Cuba

Between 1990 and 1995, the incidence of cancer in males decreased by a 1.3% average percent change per year in Cuba. While from 1996 to 2003, there was a 0.8% increase in average percent change annually in men. The age-adjusted rate (standard world population) per 100,000 (AAR) for PC incidence was 26.9 from 2001 to 2003. Now, considering 1990 to 2007, cancer mortality increased for males by a 0.28% average yearly percent shift. The AAR for PC mortality was 23.3 from 2005 to 2007 [11].

Dominica

Focusing on Dominica, no information was reported for the PC incidence rate. The percentage of PC deaths was 47.4% with the age-standardised mortality rate (ASMR) being 62.9 per 100,000 standard population for males from 2003 to 2013 [12].

French West Indies (French Guiana, Guadeloupe, and Martinique)

During 2007 to 2014, the world-standardised incidence (WSI) rate was 94.4 per 100,000 person-years for PC in French Guiana while the world-standardised mortality (WSM) rate was 16.9 per 100,000 person-years. Furthermore, WSI rates for PC in Guadeloupe and Martinique were 173.0 and 164.5 respectively. These were deemed the uppermost in the world. Additionally, WSM rates for Guadeloupe and Martinique, and French Guiana were 23.0 and 16.9 correspondingly. This mortality was greater than twice that reflected in France [13].

Grenada

From 2000 to 2009 in Grenada, there were 280 new PC cases with 258 deaths and a mortality rate of 45.3 per 100,000. Furthermore, a 30% rise in mortality (p < 0.05) was seen during this period. PC was the most prevalent cancer with the incidence rate being double the incidence rate for breast cancer, that is 64.2 for PC and 32.9 for breast cancer. From 2007 to 2013, the Grenadian PC mortality rate was 60.2 per 100,000, much greater than the Caribbean rate of 35.4 [14].

Guyana

According to the GLOBOCAN 2020 database, Guyana has 271 new PC cases per year with an ASR (world) incidence per 100,000 of 71.8. Furthermore, there are 90 PC deaths annually, with an ASR (world) mortality per 100,000 of 21.9. These rates are almost equal to the Caribbean rates of ASR (world) incidence of 75.8 and ASR (world) mortality of 27.9 [12].

Haiti

The International Agency for Research in Cancer (IARC) compiled data for 26 major cancers in 172 countries in 2002, placed in the GLOBOCAN 2002 Database. From this data for Haiti, the age-standardised (world) incidence and mortality rates per 100,000 men for PC were 38.1 and 20.0 respectively. These rates were less than those of United States Black males which were 272.0 for age-standardised (world) incidence rate and 68.1 for age-standardised (world) mortality rate for PC. Additionally, in the Caribbean, Haiti had the lowest PC mortality rate [15,16].

Jamaica

Concentrating on Jamaica, during 2003 to 2007, the crude incidence rate per 100,000 was 163 and the ASR per 100,000 per year was 188.8 for new male cancer cases. The ASR for PC was 78.1 versus 65.5 per 100,000 per year (previous report 1998 to 2002), demonstrating an increase in the incidence of PC [17]. According to the GLOBOCAN Database 2002, the age-standardised (world) mortality rate for PC in Jamaica was 22.4 much less than 68.1 for United States Black males.

The Netherlands Antilles (Aruba, Bonaire, Curacao, Saba, St. Eustatius, and St. Maarten)

A 12-year study from 1968 to 1979 for cancer incidence in the Netherlands Antilles revealed a world standardised incidence rate of 25.7 for PC from 1974 to 1979. This rate is the same in Africa and the Caribbean although less than 50% of what is discovered in Blacks in North America. From 1988 to 2014, levels of mortality have been greater in Aruba and Curacao than in the Netherlands maybe because of lower levels of health care. The largest factors were breast cancer, perinatal reasons, renal disease (only in Curacao), and vascular illnesses. No data was specifically found for PC contribution to mortality [18].

St. Kitts and Nevis

From 2001 to 2007 in Nevis, PC screening was not conducted normally, so utilisation rates are not accessible. Therefore, incidence rates are not accurately available. Furthermore, between 2002 and 2006, PC had the highest total crude mortality rate which was 30.6 deaths per 100,000. Additionally, physicians said that European and US cancer screening regulations are not suitable for the population. Future studies should concentrate on creating screening rules related to this population and techniques to encourage screening [19].

St. Vincent and the Grenadines

Razzaghi et al. [20] reported that from 2003 to 2013, the ASMR for St. Vincent and the Grenadines for PC was 58.9 per 100,000 standard population for males. Furthermore, the percentage of PC deaths was 44.5%. No data was discovered for PC incidence. Future research is required for procedures for recording new PC cases accurately and efficiently.

Suriname

According to Mans et al. [21], from 1980 through 2000 in Suriname, the average crude incidence rate for cancer was 70+/-12 overall and the sex-specific incidence rate for men was 59+/-9. One of the leading cancer sites was prostate with incidence rates rising from the age group 20-49 years on, and most significant after 50+ years. The ASMR for PC was 15.1 per 100,000 standard population for males. Additionally, the percentage of PC deaths was 18.4% [20].

Trinidad and Tobago

Concentrating now on Trinidad and Tobago, Gopaul et al. [22] analysed the data on 15,029 incident cancer cases in the Dr. Elizabeth Quamina Cancer Registry from 2008 to 2018. This revealed that the rising incidence of PC per 100,000 males ranged from 59.89 to 70.97. Furthermore, the frequency was the largest among Afro-Trinidadians with Indo-Trinidadians being second. Most of the men were diagnosed with an unknown stage of PC. Mortality increased from 16.29 to 22.00 between 2009 to 2018 except for 2008 which was 26.21 per 100,000 males yearly. Likewise, similar data from 1995 to 2009 showed the largest incidence and mortality rates for PC and lung cancer. Thus, PC remained the leading cause of cancer among men since 2008 [22]. Healthcare policies should address the large incidence of PC and concentrate on screening to ensure early diagnosis and staging.

Summary

Globally, the Caribbean has one of the largest age-standardised prostate cancer incidences which is 0.076% (76 males). Furthermore, Jamaica has 0.304% (304 males) diagnosed with Barbados having 0.16% (160 males), and Trinidad and Tobago having about 0.060% to 0.070% (60 to 70 males). The PC mortality rate in the Caribbean is the highest in the world and is the main cause of death associated with cancer in this region. As of 2020, the age-standardised mortality rate in the Caribbean is approximately three times Western Europe and North America. The data differences among the various countries in the Caribbean highlight the requirement for better cancer registries standardised for each zone.

Demographics

According to Khandwala et al. [3], PC has become more prevalent as life expectancy increases with registries globally projecting increased incidence rates of PC for men older than 65 years in approximately every country. African and Afro-Caribbean males have more aggressive PC due to delayed diagnosis, germline predisposition, and socioeconomic restraints. The Caribbean has a greater number of African descendants with more than 90% of the people from Central and West Africa. Worldwide regulations supply advice on stratifying risks and giving therapy options that are limited by accessible resources, patterns of practice, and specialised clinics. Furthermore, Black males from the Caribbean have greater age-standardised mortality rates than Sub-Saharan Africans and Africans from America [3]. The following PC demographics comprise ethnicity, socioeconomic status, and age at diagnosis in each Caribbean territory once data are accessible. Additionally, these Caribbean country profiles with available socioeconomic information are demonstrated in Table 2 [23].

**Table 2:** Caribbean country profiles with socioeconomic data Samaroo et al. [23]

Caribbean territory	Income classification	GDP per capita (US$ 2019)	Adult literacy (%)
Antigua and Barbuda	High	17,112.80	98.4
Bahamas	High	32,863.70	-
Barbados	High	18,148.20	97
Cuba	Upper-middle	8,821.80	-
Dominica	Upper-middle	8,110.60	-
Grenada	Upper-middle	10,808.70	98
Guyana	Upper-middle	6,609.60	85
Haiti	Low	1,272.50	76.5
Jamaica	Upper-middle	5,582.30	91.7
Suriname	Upper-middle	6,359.80	94.4
Trinidad and Tobago	High	17,398.00	98.7

Antigua and Barbuda

Antigua and Barbuda is a twin-island state located in the North Caribbean with a population of 96,000 with approximately 45,000 men in 2020. The census from 2013 demonstrated that 91% of the population is of African descent and in 2019, 16,918 males present between the ages of 40-74 were eligible for screening for PC in a population of elevated risk [24]. There was no clear data concerning the age of diagnosis, ethnicity, and socioeconomic status of PC. This could be a result of no national screening programme being present in 2021 although September is PC awareness month and November is men’s health month [24].

The Bahamas

The Bahamas comprises 700 islands with 29 inhabited having a population of 303,611 in the whole Bahamas and 46,994 in Grand Bahama. This 2000 consensus shows an increase of 14.8% from the 1990 census with 946 more women in the 2000 information. Approximately, 85% of the population is African, and 15% is Caucasian [9]. Furthermore, the median age at diagnosis of PC was 73 years, and at death 76 years. The census and medical records did not contain information concerning PC patients' ethnicity and socioeconomic status [9]. As such, the call for a cancer registry is greatly endorsed.

Barbados

Another Caribbean island that is independently located in the western Atlantic Ocean is Barbados. The population is approximately 270,000, with 90% African descent, 4% European origin, and less than 2% other ethnic sets [25]. According to Emtage et al. [26], the Barbados Urologic Diseases Survey database analysis was not sub-stratified for PC patients' ethnicity, which could have helped better examine the association of PC with race. Since most of the Barbadian population is African, an approximation of the disease burden can be mainly the Black group. From 1990 to 2009 in Barbados, 3066 new PC cases were discovered, and the mean age at diagnosis varied from 73.1 years to 66.2 years. Furthermore, between 2000 and 2009, the rate of high-grade (Gleason score ≥ 8) and intermediate-grade (Gleason = 7) PC increased while on the other hand, the rate of low-grade PC (Gleason score ≤ 6) decreased. This highlights the fact that there is a trend toward more aggressive disease over this decade [26].

Cuba

Focusing now on Cuba, this island contains approximately 11 million people and is separated into 14 provinces. The urban areas comprise 70% of the population while the capital city of Havana has 2 million people being the greatest concentration of the population. The majority of Cuba is Caucasian, 12% Black, and 22% Mestizos. From 1986 to 1990, PC was the third main cancer in males with around 1800 newly diagnosed cases per year. Furthermore, most men were over 65 years old at diagnosis, and over 1200 died annually [27]. Additionally, incidence and mortality increased from 1990 to 1999 [28] and a further increase was noted in incidence from 2000 to 2003 and mortality from 2000 to 2007 [11]. These trends reflect the need for further research to explain these variations and more efficient prevention and mortality reduction policies and programmes.

Dominica

According to Spence et al. [6], Dominica is an eastern Caribbean island with a population of approximately 74,000 in 2017 and no cancer registration activity. Furthermore, Razzaghi et al. [20] contend that Dominica has a mainly Black population where PC is the leading reason for death among males from 2003 to 2013, attributing to 47.4% of cancer deaths.

French West Indies

In the French West Indies, Guadeloupe and Martinique are two French territories overseas with an approximate population of 400,000 in each region. The main ethnic group is Afro-Caribbean which is 85% and has greater income inequalities, lower educational levels, and raised unemployment compared to mainland France [29]. On the other hand, French Guiana has a better socioeconomic status and contains immigrants contributing 30% of the population from South America and other parts of the Caribbean [30]. Furthermore, in this region, there were 78 new PC cases per year from 2007 to 2014, compared to more than 500 new cases per year in Guadeloupe and Martinique. There was no specific information regarding age at diagnosis [5]. From this analysis, there is a need for more reliable and up-to-date epidemiological data.

Grenada

According to Delpech and Haynes-Smith [31], Grenada is a tri-island nation that consists of Grenada, Carriacou, and Petit Martinique in the eastern part of the Caribbean. Furthermore, there are 104,000 inhabitants with more than 80% African descent and the rest of the population comprises Europeans and East Indians. PC is the main cause of cancer death among males but specific information concerning socioeconomic status and age at diagnosis of these patients is unknown. This is because there is only one pathology laboratory and no cancer registry [14].

Guyana

Lying between Suriname and Venezuela, Guyana is the only country within South America that speaks English as its first language. The main ethnicities are influenced by indentureship with 44% East Indian and slavery with 30% African. Furthermore, Indigenous Amerindians are 9% with the rest of the people being Chinese, European, or mixed [32]. This country is categorised as an upper middle-income nation [23] with Afro-Guyanese being 65% of PC cases and most males diagnosed being over 70 years [32].

Haiti

Concentrating now on Haiti, this Caribbean island is joined to the Dominican Republic in its eastern part with more than 11 million people and 95% African descent. Furthermore, this nation has poor resources, low income, and an impoverished population [33]. More research is required regarding the age at diagnosis of PC cases since no updated data was found.

Jamaica

Another Caribbean island, Jamaica, has 2.7 million people with the main ethnic group being 91.2% African of the overall population and 6.2% being mixed. Additionally, this island is ranked as a lower middle-income economy and has the greatest PC incidence rate worldwide of 304 per 100,000 reported by Glover in 1998 [34]. Future studies are needed to ascertain the age at diagnosis of PC cases because no updated information was discovered.

Suriname

According to Mans et al. [21], the Republic of Suriname is in the Northeast of South America. The population is about 531,000 with various ethnic groups consisting of Creoles, Javanese, Hindustanis, Maroons, Chinese, Europeans, Middle Eastern, and Brazilian people [35]. From 1980 to 2000, the highest PC incidence was seen in Creoles (6.4 per 100,000) followed by Hindustanis (0.8 per 100,000), and then Javanese (0.5 per 100,000) per year. Furthermore, most of the new PC cases were in the age group 50+ years [21].

Trinidad and Tobago

Another Caribbean country is the Republic of Trinidad and Tobago which has about 1.4 million inhabitants. Ethnic diversity consists of 37.01% East Indian, 31.76% African, 23.52% Mixed, <1% Chinese, Syrian/Lebanese, and Caucasian [22]. Additionally, the World Bank ranks this country as high-income because of its petrochemical assets, but the International Monetary Fund (IMF) as a developing nation [36]. Further, the mean age at PC diagnosis is 72.6 years, with those of East Indian ethnicity at 70.2 years versus those of mixed at 72.3 years or African at 73 years [36]. The significant issue of the great influx of Venezuelan immigrants could affect the dynamics of the PC population.

Summary

Generally, there were clear data for nine Caribbean countries regarding PC demographics with significant points of concern for ethnicity, socioeconomic level, and age at diagnosis. The Bahamas had the lowest age at diagnosis which was 50+ years whereas most of the Caribbean countries had an approximate age at diagnosis being >65 years. Furthermore, the influence of socioeconomic status was not ascertained. The main ethnic group was 80-95% African in the Caribbean except for Trinidad and Tobago which was 31.76% African and 37.01% East Indian, and Guyana was 30% African and 44% East Indian. This situation highlights that more data are needed on age at diagnosis and socioeconomic level among the various ethnicities to better understand the demographic causes of PC.

Clinicopathology

Anatomically, the prostate gland is positioned at the bottom of the penis in the pelvis, inferior to the bladder, and anterior to the rectum. Furthermore, the tissue is mostly glandular, creating an alkaline secretion that comprises approximately 25-30% of the seminal fluid. Changes within the basal or luminal cells are thought to give rise to malignancy. This can occur from chronic inflammation which is believed to enable malignant malformation. Malignancy from the gland is categorised as adenocarcinoma with the widespread metastatic areas being the skeleton and the lymph nodes that drain in the region. Additionally, malignant neoplasia commences with prostatic intraepithelial neoplasia (PIN) and/or atypical small acinar proliferation (ASAP) and develops into localised malignancy. Subsequently, continues to advance cancer with invasion locally, and lastly to elaborate metastatic illness [37].

PC diagnosis and risk stratification have been founded only on prostate-specific antigen (PSA) levels, clinical stage, and Gleason score until presently. As PC is considerably heterogeneous, the Gleason score utilises the addition of scores from two different histological sites. Clinical scientists use these variables to create nomograms and risk calculators. Some of these popular prognostic tools include Partin tables, Cancer of the Prostate Risk Assessment, and the Memorial Sloan Kettering Cancer Center nomogram. These variables and tools are insufficient to predict the illness course [37].

Antigua and Barbuda

According to Rhudd et al. [24], there is no national protocol regarding abnormal PSAs as the patients are referred to public or private Urologists depending on the discretion of the physician. Most prostate biopsies are done privately with image-guided and transperineal ones not accessible. Histopathology evaluation is supplied by pathologists locally at Mount St. John’s Medical Centre (MSJMC) and immunochemistry can be sought at Florida centres. As a result of these reasons, there is limited data concerning clinicopathology for PC patients.

The Bahamas

Jones et al. [38] report that from 2004 to 2016 in the Bahamas, there were 390 males with PC confirmed by biopsy. Out of these patients, 216 were recently diagnosed with a non-metastatic illness and node negative. Afro-Caribbean men were 205 and Caucasian 11 (5.1%) males. Regarding stage, 53% were non-malignant clinically (T1), 43% (T2) were malignant clinically restricted to the prostate, and 4% (T3) were outside of the prostate clinically. PSA varied from 1.6 to 400, the median was 18.0 and the mean was 31.3. Additionally, a higher T stage (Kendall’s Tau-b, p = 0.0012) was related to a higher Gleason grade associated with larger baseline PSA values (p = 0.019). On the other hand, the PSA was not related statistically to the T stage clinically (Kruskal-Wallis rank analysis).

Barbados

Focusing now on Barbados, Emtage et al. [26] examined the Urologic Diseases Survey database to obtain PC trends in Barbados from 1990 to 2009. The 3066 new PC patients were identified, and the data was investigated. At diagnosis, the mean age declined from 73.1 years in 1990 to 66.2 years in 2009. Additionally, the rate of low-grade PC (Gleason score ≤6) was lowered from 2000 to 2009. However, the rate of intermediate-grade PC (Gleason score = 7) and the rate of high-grade PC (Gleason score ≥8) increased. This demonstrates the high PC burden and the tendency towards more destructive disease over the last 10 years. The Survey database is useful in epidemiological analysis but must be carefully examined because of a deficiency in screening practice data [26].

French West Indies

According to Khandwala et al. [3], in Martinique in 2013, 84% of 473 PC patients had localised illness, and 14% had metastatic disease with positive nodes. Furthermore, Deloumeaux et al. [39] investigated 3,295 PC patients between 2008 to 2013 in Guadeloupe and found the median PSA was 8.9, and 13.6% of them had a Gleason score ≥8. A retrospective study carried out by Meunier et al. [40] demonstrated that 13% of a Martinique cohort with advanced PC locally compared to 5% in a cohort in France. This higher PC incidence in Martinique and Guadeloupe compared to mainland France could be caused by the pesticide chlordecone contaminating the ground and water which is an endocrine disruptor with an enhanced risk of PC [41].

Jamaica

In Jamaica, Coard and Skeete [42] identified 529 PC cases over six years from 2000 to 2005 at the University Hospital of the West Indies in Kingston and collected clinicopathology data from the associated histopathology forms. Only 91 of the PC patients had a PSA level ≤10.0ng/ml while 155 had PSA levels >100ng/ml. Furthermore, there were 198 moderately differentiated (37.5%) and 160 (30.2%) poorly differentiated cancers histologically. Statistical analysis of the variables demonstrated a significant moderate correlation between PSA and Gleason score. Another study conducted in Jamaica by Shirley et al. [43] investigated 99 PC patients' biopsy clinicopathology findings from 1993 to 1997 in the Pathology Department at the University of the West Indies (UWI). It was reported that the median PSA was 37 ng/ml, 63% of the patients clinically had stage T1 or T2 illness, and 60% of PC cases had a Gleason score of 8-10. Additionally, one-third of all cases got a perineural invasion, 18% had prostatic intraepithelial neoplasia, and 8% had periprostatic involvement. Both these studies reflect PC patients presenting with an advanced disease which could be a result of the absence of an organised screening protocol in Jamaica.

Further, Kampel et al. [44] reviewed 150 Jamaican-born men's records at the Memorial Sloan-Kettering Cancer Center (MSKCC) from 2003 to 2005. This study found mean age at diagnosis was 59 years, median PSA was 8 ng/ml, and Gleason scores ranged from 6 to 8-10. Organ-restricted illness accounted for 92% (75/81) by clinical stage T1-2 and Digital Rectal Examination (DRE). Out of 46 PC cases who got prostatectomies, 84% (37/44) had no nodes and 67% (31) had organ-restricted illness. These findings could be affected by bias associated with the referral of patients with a lower risk of illness.

Lastly, Aiken et al. [45] analysed the medical records of 154 urban-dwelling and 222 rural PC patients and found that rural men (72 years) were generally older than urban men (68.5 years). Additionally, rural males vs urban males had greater median PSA values, local tumour stage, greater mean Gleason scores, and more initial non-curative therapies. Since rural PC patients presented with more advanced illnesses and are mainly of African ethnicity like urban patients, this difference could be the result of variations in disease awareness, screening procedures, and gaining access to medical care.

Trinidad and Tobago

Concentrating now on Trinidad and Tobago, Mungrue et al. [46] reviewed 1250 PC patients’ epidemiological data at San Fernando General Hospital from 2002 to 2005. This study found that Africans were older than Southeast Asians (mean age 72 versus 67), had greater PSA levels (54 versus 34 ng/ml), and had greater Gleason scores (≥7 versus ≤6). Additionally, a Gleason score of 5-7 occurred in 40% of the PC patients which indicates good survival. On the other hand, 23.5% had a Gleason score of 8-10, predicting node association and a lower chance of survival.

More recently, Hosein et al. [47,16] conducted a clinicopathological examination of 546 patients’ prostate biopsies at San Fernando General Hospital from 2012 to 2014. It was found that 51.8% (283) had carcinoma, 45.6% had moderately differentiated tumors (Gleason score of 7), and 15.2% had well-differentiated tumors. Furthermore, available PSA data for 261 out of the 283 PC patients showed that 22.9% got a PSA value ≥100 ng/ml. Among these cases, 46.4% were clinical stage T2, and 36.4% were T1c. Additionally, Afro-Trinidadians (72.1%) were expectedly high-risk (63.1%), got high-grade illnesses, and had high PSA values compared to Indo-Trinidadians (25% out of the 283 PC patients).

Summary

Largely, five Caribbean countries had important information regarding PSA levels, Gleason grades, and clinical stages in PC. In the Bahamas, a greater baseline PSA was related to a higher Gleason grade associated with a higher clinical T stage. Furthermore, Barbados demonstrated a high PC burden with more aggressive PC illness from 2000 to 2009. However, the survey database must be investigated carefully because of a deficiency in screening protocol information. Compared to mainland France, Martinique and Guadeloupe had a greater PC incidence with higher PSA levels, Gleason scores, and 14% having metastatic illnesses with positive nodes in Martinique in 2013. This could have been caused by a chlordecone pesticide contamination on the ground and water. In Jamaica, there were high PSA levels, Gleason grades, and clinical stages in PC. Additionally, rural versus urban PC patients presented with more advanced diseases having higher median PSA levels, local tumour stage, and mean Gleason grades. Since both these patients were of African race, the variation may be due to a lack of patient awareness, screening protocols, and medical care access. Among Afro-Trinidadians in Trinidad and Tobago, there is a higher risk of PC illness with higher Gleason grades, higher clinical stages, and PSA levels compared to Indo-Trinidadians. Research on creating clinicopathologic techniques specific to the different territories can help in comprehending and treating PC in the Caribbean. Furthermore, immunochemistry is not available in certain countries which contributes to differences in PC profiles. This highlights the requirement for standard procedures in PC management in the Caribbean.

Genetics

Gene testing is important for PC because it can identify persons at high risk for PC who can benefit from enhanced surveillance and targeted therapy. Globally, family PC prevalence is approximately 20% and inherited PC is about 5-15%. Additionally, nearly 170 loci for hereditary PC and almost 33% of family PC risks have been detected in genome-wide association studies (GWAS). Many genes have demonstrated a robust relationship with hereditary PC which comprises BRCA1, BRCA2, ATM, CHEK2, PALB2, and Lynch syndrome which has MLHI, MSH2, MSH6, and PMS2 (Berenguer et al., 2023). Furthermore, it was found that African descendants, which account for most of the Caribbean population, had MIR151 (microRNA 151a), HPC1, and Broad11934905 A (allele-specific to African parentage) after the Human Genome Project was finished [3].

French West Indies

In the French West Indian island of Guadeloupe, where there is a predominantly African population, 498 PC cases histologically confirmed with 541 controls were utilised in a study that found rs16901979 (single nucleotide polymorphism [SNP]) sited in region 2 of 8q24. Furthermore, at rs16901979, the A allele and AA genotype were related to increased PC risks [48]. Likewise, the other French West Indian island of Martinique is among the countries globally with the greatest PC incidence and with a high incidence of onset early of PC and with familial forms. Additionally, the heterozygous germline variant HOXB13 c.853deIT was discovered in a study of 46 African PC cases carried out by Marlin et al. [49]. This study is limited due to the small sample size and use of early-onset PC patients (before the age of 51 years).

Jamaica

Focusing now on Jamaica, Dubey et al. [50] conducted a case-control study on 356 Jamaican men (162 PC cases with 194 controls) with or without sexually transmitted infections (STIs) utilising logistic regression in analyses with multiple variables. Two toll-like receptor (TLR) genes associated with SNPs were investigated and it was found that PC risk was regulated by age and IRF3_rs2304206 GG genotype in PC cases with STIs.

Another study by Dubey et al. [51] was carried out on 211 Jamaican Black men (109 PC cases and 102 controls) with or without obesity using logistic regression in multivariate analyses. Eighty-seven cytokine and chemokine-related SNPs were analysed in all the patients, and it was discovered that the CCR7 genetic variant was a significant factor related to high-grade PC risk in males with obesity. Furthermore, advanced age was an important factor in low-grade PC in men both with normal weight and obesity. Both these studies were limited because the ethnicity of the Jamaican males was not clearly stated if they were only African or mixed.

Trinidad and Tobago

According to Shea et al. [52], ELAC2 regions in 24 Afro-Caribbean PC men in Tobago which were verified histologically, were investigated with controls. The study showed the lack of ELAC2 alterations and relationships between ELAC2 polymorphisms and PC patients and controls. This led to the conclusion that ELAC2 does not give rise to increased PC prevalence in Afro-Tobagonian men. On the other hand, Okobia et al. [53] conducted a case-control study on 354 PC and 438 controls of Afro-Caribbean descent from The Tobago Prostate Cancer Survey between 2001 and 2007. Genotyping was performed and SNP rs16901979 in region 2 was found to be related to a significantly enhanced PC risk. Furthermore, there was a larger risk for SNPs rs1447295 and rs6983267 in early PC. These findings led to the conclusion that chromosome 8q24 variants are associated with the increased PC burden in Afro-Tobagonians. Likewise, Henning et al. [54] used a sample from The Tobago Prostate Cancer Survey consisting of 79 PC cases confirmed by biopsy and 87 controls. Cytokine investigations were carried out and PC cases with herpesvirus 8 demonstrated enhanced levels of interleukin-13 and interleukin-10. These results support the hypothesis that herpesvirus 8 promotes a T helper 2 immune response supporting PC creation and the continued existence of PC Afro-Tobagonians.

Summary

Overall, four Caribbean territories had published data concerning genetic markers. In Guadeloupe, rs16901979 located in region 2 of 8q24 was discovered, and in Martinique variant HOXB13 c.853deIT was found. Furthermore, the IRF3_rs2304206 GG genotype in PC cases with Jamaican males having STIs was demonstrated, and the CCR7 variant was seen to be related significantly to high-grade PC risk in obese Jamaican men. Additionally, Afro-Tobagonians showed no rise in PC prevalence due to ELAC2. However, SNP rs16901979 sited in region 2 was importantly associated with an increased PC risk, and 8q24 variants were related to the enhanced PC burden. Interestingly, herpesvirus 8 causes a T helper 2 immune response promoting PC establishment and survival in Afro-Tobagonians. Future studies are required for genetic data in the Caribbean with more significant sample amounts and cohort analysis in families. This will assist in comprehending the PC inheritance model and to analyse components in the surroundings that impact the appearance of illness.

Risk factors

Besides genetic risk factors for PC, there are recognised PC risks which include progressing age, Black ethnic background, family record of PC, insulin-like growth factors (IGF), lifestyle, diet, and environmental factors. At the time of the slave trade, many Africans were brought to the Caribbean. Considering this fact, many Caribbean nations presently demonstrate a large PC incidence [55]. Although PC screening increases Afro-Caribbean men's existence and should begin at 40 years, there are many barriers. This includes no official programmes and sticking to guidelines internationally is not known in numerous Caribbean countries [3].

Barbados

Hennis et al. [56] contend that in Barbados, men who had sexual intercourse for the first time before 16 years had an increased risk of high- and low-grade PC. Furthermore, a heightened likelihood of high-grade PC was associated with a greater number of sexual partners during life. In a literature review by Brown et al. [57], there is restricted evidence that more PC occurred in Barbadian males with less education and those married. These findings demonstrate a main concern for future studies on Caribbean PC inequalities and social determinants.

Cuba

In Cuba, a case-control study conducted by Fernández et al. [58] between 1998 to 2000 for males living in Havana diagnosed newly with PC aged up to 84 years by histology and cytology, showed that PC risk was raised with venereal disease history. Additionally, men having sexual intercourse seven times versus three or fewer times weekly demonstrated an enhanced PC risk. This supports the fact that an infectious component may be implicated, and hormonal influences associated with sexual intercourse cannot be ruled out.

French West Indies

Belpomme et al. [59] carried out a multifactorial examination utilising a transdisciplinary method to ascertain the cause of PC in Guadeloupe and Martinique. The study concluded that the increasing PC incidence cannot be associated with lifestyle changes or altered ethnographic influences. Thus, environmental factors like carcinogenic pesticides contaminating soil and water from banana estates could cause PC.

Jamaica

Focusing on Jamaica, Glover Jr. et al. [60] investigated extensively the pedigrees of 263 PC Jamaican males with 263 controls. The findings showed that a male with one first-degree PC relative was two times as probable compared to the overall population to acquire PC. Furthermore, if a person had a second-degree PC relative, there could be a statistical difference in PC risk. Another non-genetic risk factor may be a high refined carbohydrate dietary intake in Jamaican men which was reflected in the case-control study by Jackson et al. [61] on 243 PC patients with 273 controls from Jamaican urology clinics between March 2005 to July 2007.

Trinidad and Tobago

According to Mungrue et al. [46], the data for the patients admitted to the Urology unit at San Fernando General Hospital in Trinidad and Tobago between 2002 and 2005 were reviewed utilising Statistical Package for the Social Sciences (SPSS) version 16 (IBM Corp., Armonk, NY, USA). The results revealed that the average age of PC patients was 71 years which demonstrated that advanced age was a non-genetic risk factor. There were no other studies found concerning specific information on non-genetic risk factors besides ethnicity and clinicopathological features for PC in Trinidad and Tobago which were included in the other sections.

Summary

Generally, five Caribbean nations had available data regarding non-genetic risk factors. In Barbados, there were sexual factors contributing to PC. These consisted of sexual intercourse before 16 years associated with an enhanced low- and high-grade PC risk. Furthermore, a larger number of lifetime sexual partners were related to a raised high-grade PC risk. Likewise, in Cuba, sexual factors existed which comprised a venereal illness record and sexual intercourse seven times per week associated with an increased PC risk. Interestingly, carcinogenic pesticide soil and water contamination could give rise to PC in Guadeloupe and Martinique. Additionally, Jamaica had pedigree influences which included first- and second-degree relatives affecting PC risk. Further, a highly refined carbohydrate dietary intake may impact the likelihood of PC. In Trinidad and Tobago, advanced age was found to be another non-genetic risk factor. Future research is therefore needed for the non-genetic risks of PC in Caribbean countries because of the existing limited information. This will lead to more targeted surveillance and treatment.

Diagnosis and treatment

PC screening is established on serum PSA values (>4.0 ng/ml) and DRE. Following suspicion, a magnetic resonance imaging (MRI) scan is conducted normally, which suggests whether a biopsy of the prostate gland should be carried out. Afterward, the histology of the malignancy is verified, and staging is carried out through computed tomography (CT) or positron emission tomography (PET). The findings determine the treatment based on a mixture of surgery, therapy with hormones, chemotherapy, and radiotherapy [55]. The diagnosis and treatment protocols vary in the different Caribbean countries and will be discussed in the following sections.

Antigua and Barbuda

According to Rudd [24], there is no national PC screening programme in Antigua and Barbuda although September is PC awareness month and November is men’s health month. Furthermore, community clinics and private physicians offer screening with DRE and PSA, and the largest screening event is organised by Antigua Lions Club yearly. Abnormal PSA values are referred to public or private urologists since no national protocol exists. Trans-rectal ultrasound-guided (TRUS) biopsies are mainly performed in the private sector with an analysis of the histopathology by local pathologists and immunochemistry can be asked for by Florida centres. Subsequently, staging is conducted utilising X-rays and CT but no multiparametric MRI is available because of the lack of expertise and local protocols. Treatment for PC localised is mainly external beam radiotherapy (EBRT) with androgen deprivation therapy (ADT). Additionally, most of the time radical retropubic prostatectomy (RRP) is conducted privately since most public patients present at an advanced state of illness and age. PC patients requiring focal therapy, brachytherapy, and minimally invasive prostatectomy must seek these therapies abroad since these are not available locally.

The Bahamas

Concentrating on the Bahamas, in 2004, the Cancer Society started a PC awareness drive based in the community together with free PSA and DRE yearly in New Providence and Grand Bahama. With PC verified by biopsy, all these males were given treatment options according to regulations by the National Comprehensive Cancer Network. Radiation treatment and sophisticated imaging were only available privately and required paying out of their pockets [62]. Decision analytical modeling research was performed by Heijnsdijk et al. [62] on data including age and PSA values involved in PC screening tests in Nassau between 2004 and 2018 and in Freeport between 2013 to 2018. The results of this study showed that limited screening gave benefits that changed with the ages of screening and the number of tests.

Furthermore, Jones et al. [38] analysed the outcomes of PC patients with non-metastatic illness at the Cancer Centre Bahamas treated with radiotherapy, with or without androgen deprivation treatment between 2004 and 2016. The findings revealed that these outcomes are consistent with expectancies from risk-stratified regulations adopted in nations that are developed.

Cuba

In Cuba, González [63] reported that early finding is a tactical part of the Comprehensive Cancer Control Program (CCCP) which consists of a screening of seemingly healthy individuals, screening of persons at risk, and discovery of cases in patients with symptoms. Additionally, PSA is advised for symptomatic PC individuals, persons with a PC family record being 45 years or older, and those requesting PSA being older than 50 years. Ultramicro enzyme-linked immunosorbent assay (UMELISA) PSA, an assay created by the Immunoassay Center, is utilised to measure the total and free serum PSA.

Jamaica

According to Morrison et al. [34], PC screening is not a Jamaican national policy. Nevertheless, screening and patient education for PC are offered by the Jamaican Cancer Society (JCS) and the Jamaican Urological Society (JUS). Sufficient treatment for PC localised is accessible in the forms of dynamic observation, radical prostatectomy retropubically saving the nerves, radiation by external beam, and brachytherapy. PC that is advanced locally and those that are metastatic can be treated by therapy involving androgen deprivation through the National Health Fund. Furthermore, second-line hormonal and chemotherapy medications are accessible but expensive for a major part of the population. Although the PC treatment infrastructure is good, more specialist and advanced technological services are needed.

Martinique

Focusing on Martinique, Joachim et al. [64] performed cohort research that was retrospective and observational created on 452 PC patients in 2013 from the Cancer Registry in Martinique. The median PSA value at diagnosis was 8.16 ng/ml, DRE was conducted in 406 (93.8%), and clinical staging was done in 385 (85.2%) PC subjects. One treatment was obtained by 373 (82.5%) whereas 79 (17.5%) got active observation. However, external radiation treatment together with androgen deprivation therapy was the most regular combination of treatment among 102 (22.6%) PC patients. This detailed information is important for making urgent public health Caribbean protocols.

Trinidad and Tobago

In Trinidad and Tobago, there is a two-tiered healthcare structure and PC-associated issues contribute to a major part of urologists’ work. PSA testing is accessible widely, but no standard exists nationally. Furthermore, therapy options comprise dynamic observation, radical prostatectomy, brachytherapy, and radiotherapy by an external beam. Additionally, PC individuals have availability to chemotherapy, androgen deprivation, and palliative treatment for advanced illness management [65]. King-Okoye et al. [66] contend that the absence of knowledge of the prostate gland, PC symptoms, DRE, PSA, and norms of societal masculinity are principal barriers to seeking healthcare. Overall, there is a requirement for enhanced observation, quality of care, and models optimised for predicting rates of cancer in Trinidad and Tobago [36].

Summary

Largely, six out of the Caribbean countries had available information on the diagnosis and treatment of PC. In Antigua and Barbuda, there is no PC screening programme nationally despite September being PC awareness month and November being men’s health month. Furthermore, screening is performed with DRE and PSA, and abnormal PSA measurements are referred to urologists. TRUS is conducted primarily by the private sector with histopathology done locally and immunochemistry done abroad in Florida. Clinical staging is then carried out with X-rays and CT. Subsequently, PC is treated with EBRT, ADT, and RRP. Brachytherapy, focal therapy, and minimally invasive prostatectomy are only accessible abroad. Likewise, in the Bahamas, screening is conducted with DRE and PSA, and PC is confirmed with a biopsy. Similar treatment includes radiotherapy and ADT. Considering Cuba, UMELISA PSA was used to determine the free and total serum PSA which was a strategic component of its CCCP. Additionally, JCS and JUS offered patient education and screening for PC but no PC screening national policy. Treatments available comprised EBRT, ADT, RRP, and brachytherapy with costly second-line hormonal and chemotherapeutic drugs being also accessible. In Martinique, screening was carried out with DRE and PSA, and then clinical staging. The most frequent mixture of treatments was EBRT and ADT. Focusing on Trinidad and Tobago, PC screening with DRE and PSA, and all treatments are widely accessible. However, the main barriers to seeking medical care include a lack of education regarding PC-related issues and norms of societal masculinity.

Prostate cancer control

Responsive cancer care in the Caribbean is difficult because there is a broad spread of islands, under-funded medical care systems, and the lack of a unified strategy for cancer control. Only seven CARICOM (The Caribbean Community and Common Market) countries have individual cancer control plans on a national level [6]. This points to the fact that there is a need for governmental and scientific organisations to provide a unified response to enhance cancer studies and observation with respect to the specific requirements of each Caribbean island [5].

Antigua and Barbuda

According to Rudd [24], every area of the path to PC care exists in Antigua and Barbuda from the awareness of the public and screening to EBRT, ADT, and institutional care. Yet some limitations are present which include the availability of prostate biopsies, radical prostatectomies, and more recent imaging techniques for clinical staging [24].

The Bahamas

In the Bahamas, Roberts et al. [67] discovered a prosperous PC screening Grand Bahama clinic that is community-based which found 40 patients with a positive-predictive value of 89%. These cases were then advised by a urologist and allowed standard care. Furthermore, Heijnsdijk et al. [68] analysed the PC screening plans in a populace at high PC risk with minimal resources in Nassau and in Freeport, Bahamas, and found that the PSA screening programmes have a more positive harm-to-benefit ratio than countries that are at a high-income level. Additionally, Jones et al. [38] investigated the outcomes of PC cases at a Bahamian cancer center from diagnosis and found that these outcomes were consistent with the expected results from the guidelines of the stratification of risk followed in countries that are developed. Thus, there are encouraging projects in place in the Bahamas.

Cuba

Focusing on Cuba, Martín et al. argued that the quality of the data was poor to international ideals although the National Cancer Registry stated that there was an enhancement of data quality indexes between 1986 to 1990. On the other hand, the results of the Registry appeared to be a beneficial tool for assessing the burden of cancer and more significantly controlling and ultimately adjusting the assignments of the National Cancer Control Program [15].

Jamaica

According to Morrison et al. [34], PC accounted for a large part of Jamaican clinical practice, and the number of patients to the number of accessible urologists is high, restricting sufficient medical care. PC screening is not a national Jamaican policy, but the Jamaica Cancer Society and the Jamaica Urological Society promote the education of patients and screening. Furthermore, Morrison et al. [66] conducted a retrospective evaluation of 1117 patients at the Jamaica Cancer Society screening clinic from 1995 to 2005. This study demonstrated that older men tend to screen for PC more than younger men in Jamaica, limiting the age range for screening. Additionally, Morrison et al. [67] performed a cross-sectional evaluation of 55 males who went for PC screening at the Jamaican Cancer Society in 2014. It found that Jamaican men have average PC knowledge and a positive view towards PC prevention actions and PC screening. However, altering modifiable risk factors in practice is inadequate. Aiken and Eldemire-Shearer [1] contend that future studies are needed to ascertain locally modifiable risk factors so that preventive tactics can be introduced at the populace and individual levels. Furthermore, efficient secondary preventive techniques like mass PC screening should be contemplated as soon as possible to bring this illness under control.

Martinique

Looking at Martinique, Joachim et al. [64] carried out a cohort research on 452 PC cases from the Martinique Cancer Registry in 2013. Information was obtained concerning demography, PSA value at diagnosis, Gleason score, clinical stage, and patterns of healthcare which were then evaluated. This study supplied detailed information concerning diagnosis quality and management of PC cases. Additionally, the outcomes of the PC patients and their quality of life would be analysed, utilising a digital platform for case-reported measures of outcomes. In doing so, PC control can be achieved in Martinique, and this data is central for the creation of high-significance public health methods for the Caribbean.

Trinidad and Tobago

Concentrating on Trinidad and Tobago, Persaud et al. [65] reported that PC therapy options consist of dynamic observation, radical prostatectomy, EBRT, ADT, brachytherapy, chemotherapy, and palliative care for advanced illness. Despite all these available treatment options, the main barriers to obtaining medical care include a lack of knowledge of PC symptoms, prostate gland, DRE, PSA, and norms of masculinity in society [66]. Generally, the treatment infrastructure is reasonable but there is a requirement for further finance in technology and human resources [65]. In addition, there should be improvements in surveillance, quality of care, and optimised models for foretelling cancer rates to further control PC [36].

Summary

Overall, six out of the Caribbean nations had accessible data on systems for PC control. Screening programmes for PC were available in Antigua and Barbuda, The Bahamas, Jamaica, and Trinidad and Tobago. In Cuba, the quality of the data from the National Cancer Registry was poor according to international standards but this information provided a useful tool for evaluating the PC burden and eventually, this will lead to making the correct adjustments in the tasks of the National Cancer Control Program. Interestingly, Martinique utilised a digital platform for evaluating the quality of life and the outcomes of PC cases. This data is important for developing significant public health systems in the Caribbean. Although the treatment structure is adequate in Trinidad and Tobago, there should be further investments in human resources and technology like enhanced cancer rate models to increase PC control. Systems of PC control must be distinctly recorded, and observance of these regulations must be assessed frequently.

## Conclusions

Thus, it is fair to conclude that the objectives of this literature review were fully met which were to evaluate the dynamics of PC in the Caribbean and ascertain areas where further improvements are required. The analysis revealed the variations in incidence, etiology, management, and mortality among the Caribbean nations. Those countries with few resources are burdened by more advanced illnesses with worse PC outcomes. Furthermore, territories with national cancer registries appear to have an improved methodology for PC management. Additionally, thorough PC control comprises prevention, early detection, diagnosis and treatment, as well as palliative care. In this regard, education on palliative treatment, directed at our healthcare providers together with academic programmes can enhance illness outcomes. The significance of this review is demonstrated by the fact that it provides important initial support to researchers, administrators, and planners for healthcare who are concerned with the PC population in the Caribbean of different races, immigrants, socioeconomic positions, environmental factors, and societal norms of masculinity. Further, this is a chance for epidemiological analyses to provide greater insights into the PC situation.

Future interventions should consist of the establishment of national cancer registries, screening programmes, promotion of PC awareness, prevention strategies, and treatment standardisation in Caribbean countries where these are not available. In addition, cancer registries should be optimised technologically and there should be robust standardised monitoring and recording of PC outcomes to accurately reveal the PC status in the Caribbean. Further studies are required to concentrate on prevention strategies and standardisation of treatment methods to direct improved surveillance with predictable enhanced patient outcomes.

## Appendices

The specified Caribbean population comprised Antigua and Barbuda, The Bahamas, Barbados, Cuba, Dominica, French West Indies (French Guiana, Martinique, and Guadeloupe), and, Grenada, Guyana, Haiti, Jamaica, The Netherlands Antilles, St. Kitts and Nevis, St. Vincent and the Grenadines, Suriname, and Trinidad and Tobago.
